# Loss of stimulator of interferon genes (STING) promotes accumulation of cholesterol and triglycerides throughout life in mice

**DOI:** 10.1186/s40659-025-00624-3

**Published:** 2025-07-02

**Authors:** Ian Riquelme, Daniela Carrillanca, Camila Sánchez-Pérez, Andrea Monterroza, Bairon Hernández-Rojas, Gonzalo Riadi, Gonzalo I. Cancino, Paola Murgas

**Affiliations:** 1 Escuela de Medicina, Facultad de Medicina y Ciencia, Universidad San Sebastián, Sede de la Patagonia, Puerto Montt, Chile; 2 Facultad de Medicina, Instituto de Inmunología y Parasitología, Universidad Austral de Chile , Valdivia , Chile; 3 Program in Sciences Mention Modeling of Chemical and Biological Systems, School of Bioinformatics Engineering, Center for Bioinformatics, Simulation and Modeling, CBSM, Department of Bioinformatics, Faculty of Engineering, University of Talca, Talca, Chile; 4 Center for Bioinformatics, Simulation and Modeling, CBSM, Department of Bioinformatics, Faculty of Engineering, University of Talca, Talca, Chile; 5 Laboratorio de Neurobiología, Facultad de Ciencias Biológicas, Pontificia Universidad Católica de Chile, Santiago, 8331150 Chile; 6 Center for Integrative Biology, Faculty of Sciences, Universidad Mayor , Santiago, Chile

**Keywords:** STING, Lipid metabolism, Triglycerides, Cholesterol, Aging, Adipose tissue, Hepatic steatosis, Metabolic control, Innate immunity

## Abstract

**Background:**

The Stimulator of Interferon Genes (STING) pathway is pivotal in innate immunity, facilitating the detection of cytosolic DNA and initiating type I interferon-dependent responses. In addition to its immunological roles, STING has been increasingly associated with metabolic regulation, since research indicates that its inhibition can diminish inflammation, lipid accumulation, and tissue damage in obesity and other metabolic disorders. The findings have prompted the suggestion of STING inhibition as a viable treatment approach for metabolic illness. Nonetheless, the physiological function of STING in lipid homeostasis under normal settings remains largely unexplored, as does the impact of its absence on metabolism throughout various life stages in the absence of disease. This information deficit is crucial, particularly in light of the increasing interest in the long-term pharmacological suppression of STING.

**Results:**

To examine the function of STING in lipid metabolism during physiological, non-pathological conditions throughout the lifespan, we assessed WT and STINGKO mice at various ages and discovered that STING deficiency results in a consistent increase in body weight, independent of alterations in locomotor activity or food consumption. STINGKO mice exhibited markedly increased circulation levels of triglycerides and total cholesterol. Histological and morphological analysis demonstrated augmented fat accumulation in adipose and hepatic tissues, despite the lack of nutritional or genetic metabolic stress. These findings indicate a crucial function for STING in the control of lipid homeostasis across the lifespan.

**Conclusions:**

In contrast to earlier research conducted under pathological conditions, our findings indicate that the total absence of STING expression in healthy contexts leads to heightened lipid accumulation in tissues and blood. These findings underscore an unforeseen function of STING as a modulator of lipid metabolism in the context of longevity. They caution against the prolonged use of STING inhibitors, as chronic STING suppression may lead to detrimental metabolic effects. This study offers new insights into the non-immune roles of STING, indicating its significance in preserving metabolic equilibrium throughout the lifetime.

## Introduction

Recent evidence demonstrates that lipid metabolism is influenced by molecules commonly linked to the immune system [1–3]. In adipose tissue and liver, various molecules are expressed that regulate immune responses and lipid metabolism [3, 4]. The stimulator of interferon genes (STING) is a crucial protein implicated in inflammation and is linked to lipid metabolism [5–8]. STING is expressed in pre-adipocytes, adipocytes, and macrophages in adipose tissue [9–11]and is prominently expressed in Kupffer cells, a specialized macrophage type in the liver; however, its presence in hepatocytes is subject to debate. Activation of STING due to cellular damage and the release of pro-inflammatory cytokines has been observed in metabolic disorders, including obesity, insulin resistance, type II diabetes, nonalcoholic steatohepatitis (NASH), and various dyslipidemias [5, 6, 8, 10, 13].

STING, a transmembrane protein located in the endoplasmic reticulum (ER), is involved in the detection of cytosolic nucleic acids to trigger the immune response [15, 16]. The principal activator, the enzyme cyclic GMP-AMP synthase (cGAS), synthesizes cyclic GMP-AMP (cGAMP) upon detecting cytosolic double-stranded DNA (dsDNA) from pathogens or damaged self-DNA (nuclear/mitochondrial) [15, 16]. cGAMP interacts with STING, initiating a signaling cascade in which the STING-cGAMP complex translocates to the Golgi and recruits Tank-binding kinase 1 (TBK1) [15, 16]. TBK1 phosphorylates STING, resulting in the recruitment and phosphorylation of the Transcription Factor Interferon Regulatory Factor 3 (IRF3), which subsequently dimerizes and activates type I interferon (IFN) genes in the nucleus [15, 16]. Additionally, TBK1 phosphorylates the NF-κB inhibitor, which results in the release of NF-κB, thereby promoting the production of pro-inflammatory cytokines such as IL-1β, IL-6, and TNF-α [15, 16]. In metabolic diseases, STING signaling is activated by cellular damage, such as altered cytosolic dsDNA, which drives inflammation that exacerbates metabolic dysfunction [11–18]. The long-term effects of STING absence or inhibition on lipid metabolism in non-pathological contexts have yet to be investigated.

In STING^gt^ mice, which express STING but lack downstream signaling, exposure to a high-fat diet (HFD) for 12 weeks resulted in a decrease in obesity, accompanied by reduced circulating lipids and body weight [18]. Another investigation involving STING^gt^ mice indicated that in non-alcoholic fatty liver disease (NAFLD), these subjects displayed decreased hepatic steatosis, inflammation, and fibrosis [19]. In STING^gt^ mice with induced NASH on a methionine- and choline-deficient diet (MCD), there was a reduction in steatosis, fibrosis, and hepatic inflammation, as well as lower triglyceride and cholesterol levels [9]. In human hepatocyte cell lines treated with free fatty acids for 24 h, partial STING inhibition through siRNA resulted in decreased levels of pro-inflammatory cytokines and reduced lipid accumulation [20]. Furthermore, in Kupffer cells derived from obese WT and STING^gt^ mice, pre-treatment with an NF-κB transcription factor inhibitor, which acts as a downstream effector of STING, resulted in a reduction of pro-inflammatory cytokine production [9]. STING knockout (STINGKO) mice subjected to 12 weeks of an HFD demonstrated enhanced insulin sensitivity and glucose tolerance [21]. In IRF3KO mice, which absence IRF3, a downstream transcription factor of STING, there was an increase in macrophage infiltration into adipose tissue, leading to inflammation [18]. The findings collectively suggest that STING-deficient mice display diminished steatosis, fibrosis, inflammation, and other metabolic effects when exposed to an HFD [17, 18]. However, the impact of STING inhibition in non-pathological conditions or throughout the lifespan has yet to be investigated.

Investigations into STING and its downstream signaling in lipid metabolism-related diseases have predominantly concentrated on short-term studies employing pharmacological inhibition or STING^gt^/STINGKO mice over limited time frames, utilizing HFD [18–22]. The long-term consequences of STING deficiency and its involvement in non-pathological conditions are largely unexplored. Evaluating STING as a therapeutic target for metabolic diseases requires an understanding of its impact on metabolic regulation over time, independent of obesity or metabolic disorders.

This study investigates the effect of constitutive STING deficiency on fat metabolism throughout the lifespan. We assessed physical, morphometric, histological, and biochemical parameters to investigate alterations in fat metabolism in mice lacking STING. Wild Type and STINGKO mice at young, adult, and old ages, maintained on a standard chow diet, were evaluated for body weight, muscle strength (via dynamometer and hanging test), and locomotor activity (using the Open Field test). STINGKO mice demonstrated elevated body weight across all ages; however, no significant differences were noted in food consumption, muscle strength, or locomotion. Morphometric and histological analyses indicated that STINGKO mice exhibited enlarged adipose and hepatic tissues, accompanied by increased fat accumulation. Additionally, circulating triglyceride and total cholesterol levels were significantly increased in STINGKO mice at all ages. The findings suggest that STING is involved in lipid metabolism across the lifespan, and its deficiency leads to increased fat accumulation. This study highlights the need for additional research into the chronic reduction or inhibition of STING as a potential therapeutic approach for lipid metabolism-related disorders.

## Methods

### Experimental animal groups

The C57BL6J (B6) Wild Type (WT) and STING Knock Out (STINGKO) mice, both derived from the same B6 background, were acquired from The Jackson Laboratory, USA. All mice had ad libitum access to water and were fed Lab Diet 5P00 Prolab RMH 3000. All mice were housed in a room with a 12-hour light-dark cycle, with the temperature regulated between 20 and 22 °C. Male WT and STINGKO mice were utilized, excluding females, to eliminate results influenced by hormonal factors. The subjects were categorized by age: young (1 to 4 months), adult (10 to 15 months), and old (21 to 24 months) [23, 24]. The Bioethics and Biosafety Committee of Universidad Mayor, IACUC ID 052018, granted approval for animal maintenance and all procedures conducted in this study.

### Body weight assessment

Body weight was assessed using a weight scale on live male WT and STINGKO mice of established ages, sourced from maintenance colonies, some of which were utilized for additional analyses in this and forthcoming articles. In a 15-week longitudinal study, young males (1–3 months old) of both genotypes were weighed every week.

### Food consumption assessment

Food consumption was assessed weekly by supplying 200 g of food per four-animal cage and weighing the leftover food after a period of seven days. The total consumption (delta) was divided by 4 to calculate the consumption per animal. This procedure was conducted weekly over a period of 15 weeks for longitudinal measurement purposes.

### Locomotor tests

*Forelimb grip strength*. The forelimb grip strength for each experimental group was assessed using the previously described technique [25]. Each animal was positioned on a grid linked to a dynamometer that quantified the strength of its forelimbs. The operation was conducted thrice for each animal, and the mean force was calculated. We estimated the ratio of grip strength to body mass for each animal to evaluate the correlation between these two variables.

#### Hanging test

We moreover assessed the comprehensive physical strength of each trial group utilizing this exam [26]. Mice were positioned at the center of a grid and maintained their grip with their forelimbs for 180 s. The number of falls, restricted to 10, was registered. An analysis similar to the Kaplan-Meier curve was used to determine the time until a fall occurs.

#### Open field test (OFT)

This test was employed to evaluate anxiety-like responses and locomotor activity in animals [27]. In summary, we confined the animals into a box measuring 40 cm (length) x 40 cm (width) x 40 cm (height). Subsequently, we documented and assessed the total distance traversed (y-axis) in meters for each experimental animal group during a duration of 10 min utilizing ANY-maze v6.34 software.

*Rotarod test*. This assessment measured performance in response to imposed exercise [26]. Animals were positioned on a rod, which initially moved at a steady speed of 4 rpm; once all the mice were in place, the rod accelerated from 4 rpm to 40 rpm over a duration of 300 s. Subsequently, we measured the latency time, defined as the duration (in seconds) that the mouse sustains its equilibrium on the rod during the test.

### Morphometric and histological analysis

Following the weighing of the mice, they were sedated in a chamber containing 3% isoflurane for tissue collection. Subsequently, they were exsanguinated via heart puncture using 25 ml of saline solution per mouse. The colon, gonadal adipose tissue, and liver were surgically excised.

*Colon length*. The colon segment of each animal from the specified experimental groups was excised, and its length was quantified using a ruler (centimeters).

Adipose and hepatic tissue. The gonadal adipose tissue, classified as a form of visceral fat in mice [28]was excised (except the testicles), weighed, and measured for length and width [29]. The segment of this tissue most distant from the testis was removed for histological examination. Subsequently, Eqs. 1 and 2 were employed to get an area measurement for abdominal adipose tissue and liver. Initially, the weight (in grams) (W) was multiplied by the product of length and width (in centimeters) (A1) (Eq. 1). An area of abdominal adipose tissue was obtained by normalizing this new value (A1) with regard to each animal’s weight (AW) (grams) (Eq. 2).

1 $$\mathrm{P}(\mathrm{~W}) \times \text { length }(\mathrm{cm}) \times \text { width }(\mathrm{cm})=\mathrm{A} 1\left(\mathrm{~g} \times \mathrm{cm}^2\right)$$

2 $$\text { Normalization }=\frac{\mathrm{A} 1\left(\mathrm{~g} \times \mathrm{cm}^2\right)}{\mathrm{AW}(\mathrm{~g})}$$

The distal end (0.5 × 0.5 cm) of the extracted gonadal adipose tissue, located furthest from the testes, was fixed in 4% paraformaldehyde (4% PFA) (Sigma) for 24 h at 4 °C. Subsequently, it was embedded in Tissue-Tek O.C.T. compound for cryosectioning and later stored at -80 °C until ready for use. The cryostat LEICA CM1950 was employed to acquire histological sections with a thickness of 12 μm. The sections were then stained with a Hematoxylin & Eosin [H&E] (Sigma) solution. Five sections from each adipose tissue sample of each animal were photographed, capturing 10 images per field of view at 40x magnification with a Nikon ECLIPSE E200LED MV R bright-field microscope. Analysis of adipocyte area was carried out utilizing ImageJ software.

The caudate lobe was excised from each animal after the liver was weighed and measured. Dissection and fixation of this lobe were conducted in 4% PFA (Sigma) at 4 °C for 24 h. This was followed by histological processing using the Thermo Microm STP120 tissue processor and embedding in a Thermo HistoStar paraffin block. The Leica RM 2125 RTS microtome was employed to acquire sections with a thickness of 3 μm. Subsequently, the sections were stained with H&E (Sigma) solution. Images from an area of 120 μm² were segmented into quadrants to assess the presence of steatosis. Consequently, the presence (score of 1) or absence (score of 0) of fat in each quadrant was calculated, and a final addition score was determined, with a maximal score of 4 (steatosis in all four quadrants).

### Biochemical parameters

The animals in each experimental group, after a 4-hour fast [30]were restrained to obtain blood from the tail using a scalpel. 50 μm of this whole blood sample were used for glucose measurement employing an Abbott Freestyle Optium glucose meter with Abbott Freestyle Optium test strips (mg/dl). The residual volume of the whole blood sample (200 to 300 µl per animal) was centrifuged at 2500 rpm for 15 min at ambient temperature, utilizing an Eppendorf 5415 C centrifuge Thermo Scientific™ Fresco™ 17, and the supernatant (serum) was extracted. Total cholesterol and triglycerides were quantified from this supernatant utilizing 100 µl of blood with LiquiColor^®^ commercial kits, both sourced from the same vendor. Absorbances were quantified utilizing a Tecan Infinite^®^ 200 PRO NanoQuant spectrophotometer at a wavelength of 546 nm. The next table shows the means, standard deviations (SD), and coefficients of variation (CV) for each biochemical parameter (Table 1).

**Table 1:** Mean, standard deviation, and coefficient of variation of serum glucose, triglycerides, and total cholesterol from WT and STINGKO mice at different ages

		Glucose			Triglycerides			Total Cholesterol		
Ages	Group	Mean (mg/dL)	SD (mg/dL)	CV (%)	Mean (mg/dL)	SD (mg/dL)	CV (%)	Mean (mg/dL)	SD (mg/dL)	CV (%)
Young	WT	175.8	17.7	10.0	166.4	13.6	8.1	64.0	2.4	3.7
	STINGKO	175.1	21.7	12.4	209.3	9.2	4.42	130.0	5.2	4.0
Adult	WT	149.0	19.7	13.2	166.0	16.5	10.0	69.37	14.2	20.5
	STINGKO	161.0	18.3	11.3	221.4	22.5	10.1	141.3	10.3	7.3
Old	WT	134.8	10.3	7.6	162.4	3.6	2.23	65.3	10.4	15.9
	STINGKO	146.43	26.5	18.13	222.5	21.1	9.49	136.5	21.0	15.4

### Data analysis

Data were analyzed using GraphPad PRISM v8.0 (GraphPad Software, San Diego, CA, USA). The Shapiro-Wilk test determined data normality. A one-way Analysis of Variance (ANOVA) was used to compare three or more independent groups. To compare two independent groups, an unpaired Student’s t-test or the non-parametric Mann-Whitney U test were performed. All data are shown as mean ± SEM. Statistical significance was determined at *p ≤ 0.05, **p ≤ 0.01 and ***p ≤ 0.001.

## Results

### STINGKO mice weigh more throughout their lifespan without increasing food intake

**Fig. 1:**
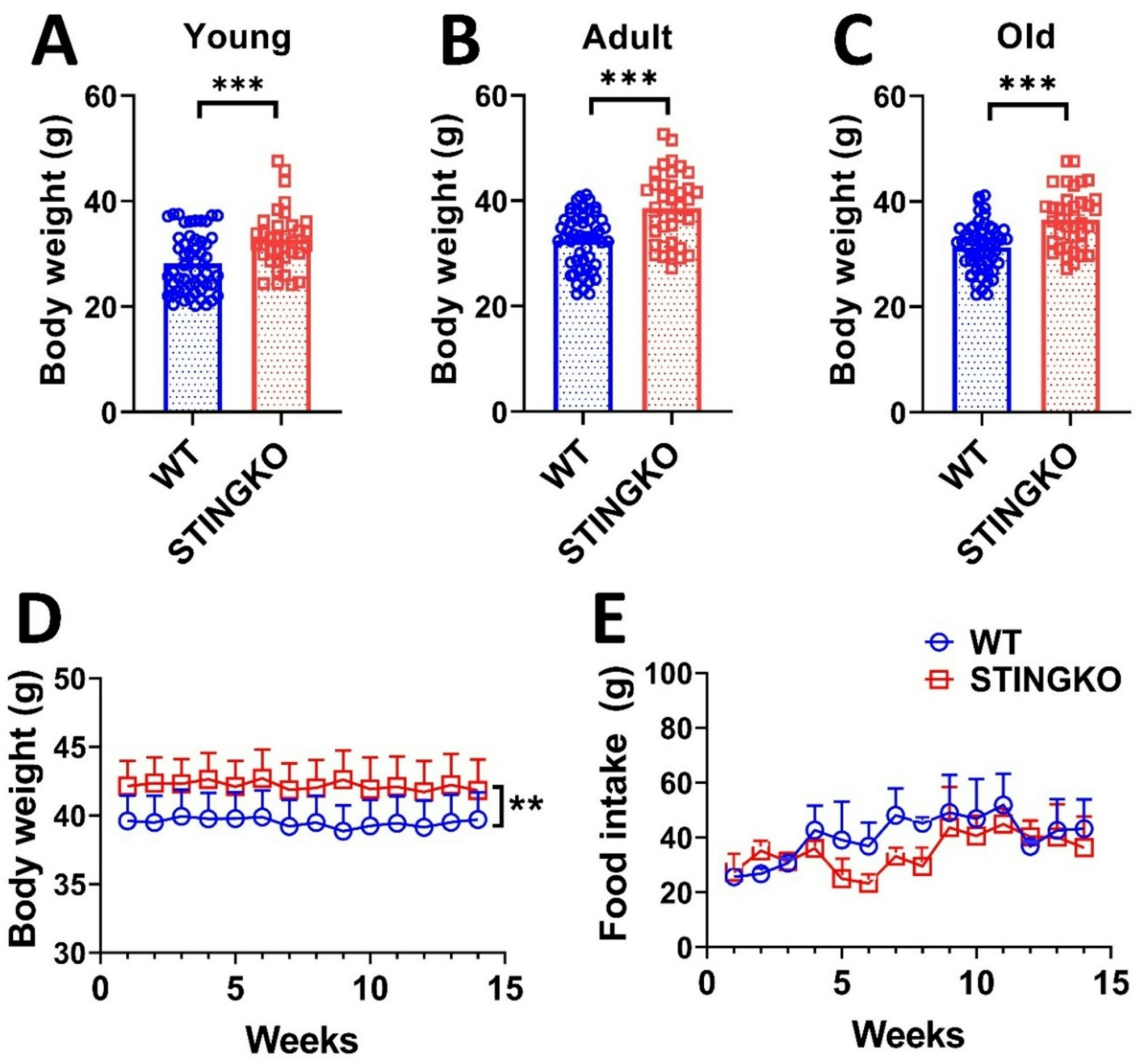
STING deficiency results in elevated body weight despite food consumption and age. Body weight was assessed in **A**) young (1–4 months), **B**) adult (10–15 months), and **C**) old (21–24 months) WT (blue) and STINGKO (red) mice. The experimental groups comprised WT mice at young age (*n* = 39), adult age (*n* = 54), and old age (*n* = 55), in addition to STINGKO mice at young age (*n* = 32), adult age (*n* = 37), and old age (*n* = 38). Data are expressed as mean ± standard error of the mean (SEM). Statistical significance was assessed using an unpaired Student’s t-test, with ***p < 0.001. **D**) Longitudinal evaluation of body weight in WT (*n* = 15) and STINGKO (*n* = 16) mice commencing at 3 months of age throughout a duration of 15 weeks. Food consumption was measured at 15 weeks in WT (*n* = 15) and STINGKO (*n* = 16) mice, commencing at 3 months of age. Data are expressed as mean ± SEM and analyzed via one-way ANOVA, with **p < 0.01

While it has been demonstrated that the absence of STING expression inhibits changes in lipid metabolism and body weight in animal models of obesity [11, 12, 17]it remains unassessed whether STING influences body weight under physiological conditions. To evaluate potential variations in body weight between WT and STINGKO mice during their lifespans, weight measurements were collected at several age categories: young (1 to 4 months), adult (10 to 15 months), and old (21 to 24 months). WT mice demonstrated an average weight of 26 ± 3.76 g (*n* = 39) during the young age, 32.41 ± 0.68 g (*n* = 54) in adult, and 32.87 ± 0.78 g at the old stage (*n* = 55). Conversely, STINGKO mice exhibited weights of 38.15 ± 0.99 g (*n* = 32), 39.07 ± 1.12 g (*n* = 37), and 38.13 ± 0.86 g (*n* = 38) across the corresponding age categories (Fig. 1A-C). The results indicate that STINGKO mice consistently exhibit a considerably greater body weight than WT mice throughout all examined age groups (Fig. 1A-C). Following that, we analyzed food consumption to determine the underlying reason for the weight disparity between WT and STINGKO mice over time. Young mice (3 months old) were observed for weekly weight and food intake over a duration of approximately 4 months, resulting in the subjects being 7 months old by the end of the study. An average weight difference of approximately 5 g was observed between WT and STINGKO mice in the body weight analysis (p-value < 0.05) (Fig. 1D). Moreover, the analysis of food consumption revealed no significant variations between the two groups (Fig. 1E). The data suggest that variations in food consumption cannot be explained for the weight differences seen between WT and STINGKO mice at various ages.

### STING deficiency does not diminish locomotor activity in mice during aging

**Fig. 2:**
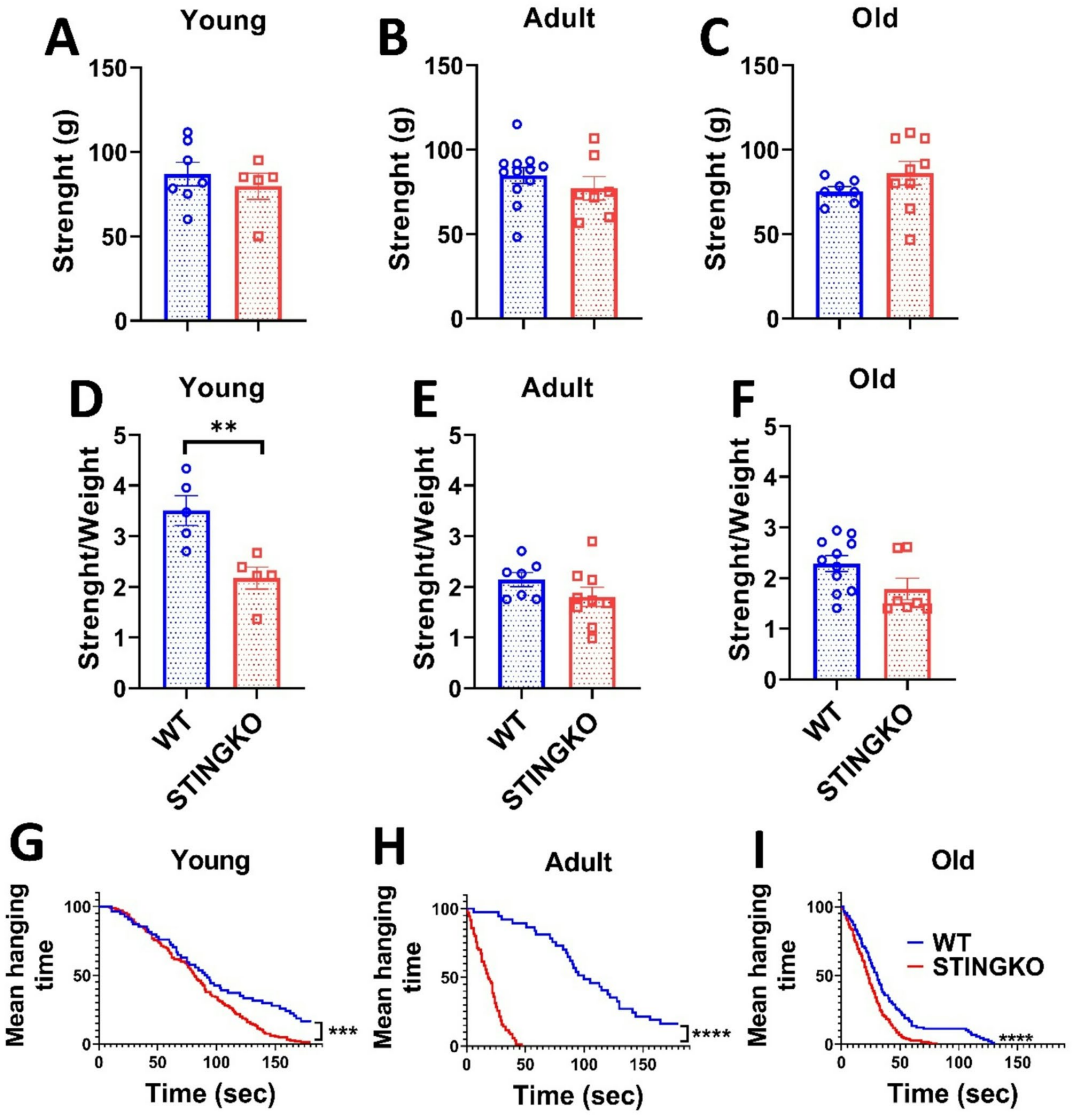
STING deficiency does not influence strength across various ages. Strength assessment in **A**) young (1–4 months), **B**) adult (10–15 months), and **C**) old (21–24 months) WT (blue) and STINGKO (red) mice. The experimental groups included WT mice at young age (*n* = 7), adult age (*n* = 12), and old age (*n* = 7), alongside STINGKO mice at young age (*n* = 5), adult age (*n* = 7), and old age (*n* = 9). Data are expressed as mean ± standard error of the mean (SEM). Statistical significance was assessed using an unpaired Student’s t-test, revealing no statistically significant differences. The ratio of strength to weight in WT and STINGKO mice at **D**) young, **E**) adult, and **F**) old ages. The experimental cohorts included WT mice at young age (*n* = 5), adult age (*n* = 7), and old age (*n* = 11), with STINGKO mice at young age (*n* = 5), adult age (*n* = 10), and old age (*n* = 7). Data are expressed as mean ± SEM. Statistical significance was assessed using an unpaired Student’s t-test, with **p < 0.01. Hanging test at **G**) young, **H**) adult, and **I**) old. The experimental groups included WT mice at young age (*n* = 8), adult age (*n* = 9), and old age (*n* = 10), along with STINGKO mice at young age (*n* = 10), adult age (*n* = 9), and old age (*n* = 8). Data are expressed as mean ± SEM and evaluated via one-way ANOVA, with significance levels of ***p ≤ 0.001 and ****p < 0.0001

Considering that STINGKO mice consistently display elevated body weight at all ages, irrespective of food consumption, in comparison to WT mice, we examined whether this disparity could be related to augmented muscle mass and, subsequently, enhanced strength. Forelimb grip strength was measured with a dynamometer to evaluate potential variations in force. The mean peak power delivered by each animal over three trials was recorded and represented (Fig. 2A-C). Significant differences in grip strength were not observed between WT and STINGKO mice, nor were any age-related effects observed (Fig. 2A-C). Additionally, a statistical analysis revealed substantial changes in grip strength related to body weight between juvenile STINGKO mice and WT mice (Fig. 2D). Nonetheless, no notable differences were detected in the adult or old cohorts (Fig. 2E-F). The data indicate that STINGKO mice probably do not show an increase in forelimb muscle mass and, thus, do not demonstrate superior strength relative to WT mice.

Then, we also evaluated physical strength using the hanging test. The number of falls for each mouse was recorded, and the average values for each experimental group were plotted (Fig. 2G-I). The number of falls increased with age in all experimental groups. At all ages assessed, STINGKO mice exhibited a higher number of falls compared to WT mice (Fig. 2G-J). These results suggest that STINGKO mice have a reduced ability to keep their grip on the hanging test bars for extended periods. This impaired performance may be attributed to either a more pronounced locomotor deficit in STINGKO mice compared to WT mice or to the potential influence of body weight on their ability to maintain grip relative to WT controls.

**Fig. 3:**
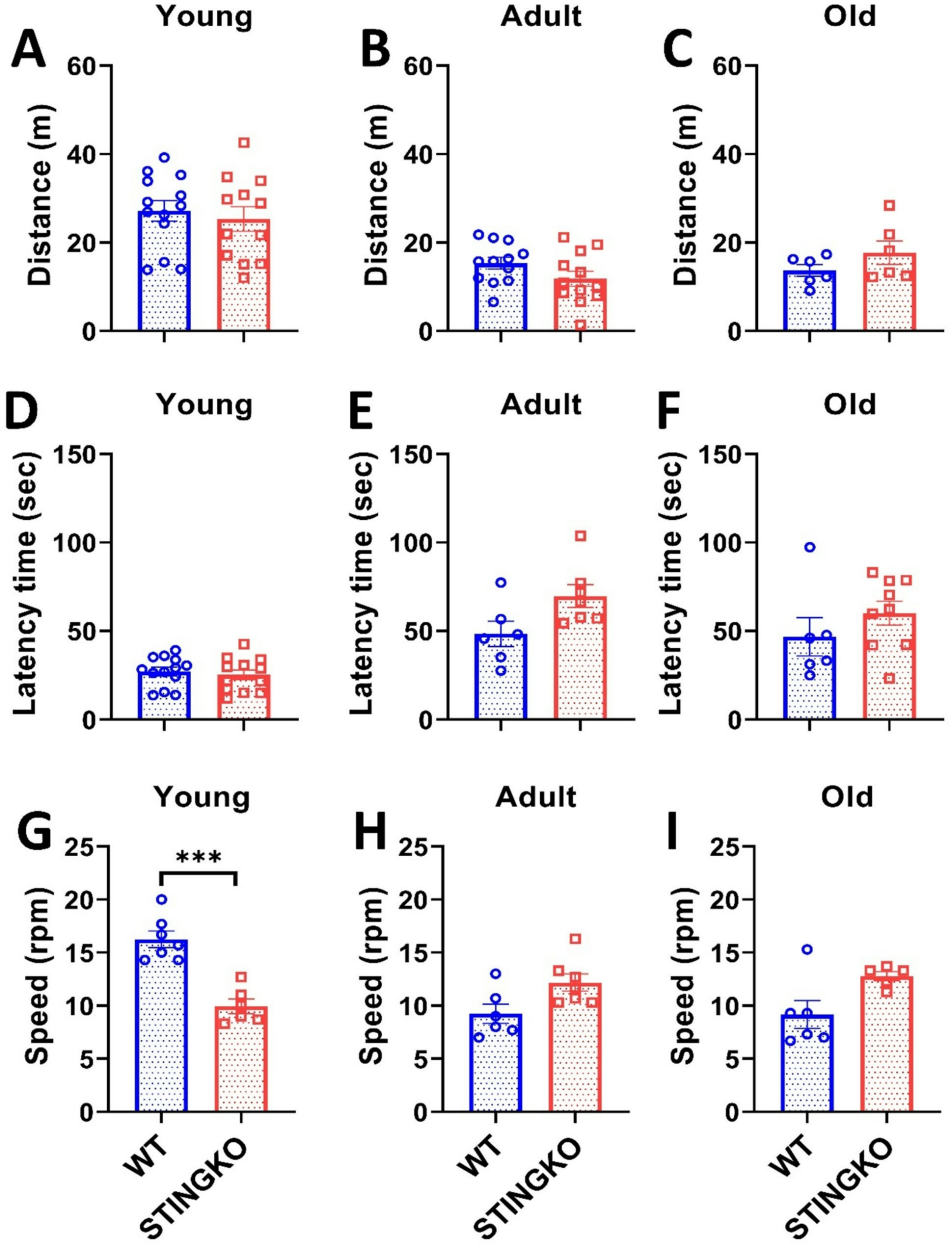
STING deficiency does not influence performance in forced or voluntary exercise across various ages. Distance traveled in the Open Field test for **A**) young (1–4 months), **B**) adult (10–15 months), and **C**) old (21–24 months) in WT (blue) and STINGKO (red) mice. The experimental groups included WT mice at young age (*n* = 12), adult age (*n* = 12), and old age (*n* = 6), along with STINGKO mice at young age (*n* = 12), adult age (*n* = 12), and old age (*n* = 6). Data are expressed as mean ± SEM. Statistical significance was assessed using an unpaired Student’s t-test, revealing no statistically significant differences. The latency period in WT and STINGKO mice at **D**) young, **E**) adult, and **F**) old stages. The experimental groups comprised WT mice at young age (*n* = 12), adult age (*n* = 12), and old age (*n* = 6), alongside STINGKO mice at young age (*n* = 12), adult age (*n* = 12), and old age (*n* = 6). Data are expressed as mean ± SEM. Statistical significance was assessed with an unpaired Student’s t-test, revealing no statistically significant differences. The speed in WT and STINGKO mice at **D**) young, **E**) adult, and **F**) old stages. The experimental cohorts included WT mice at young age (*n* = 12), adult age (*n* = 12), and old age (*n* = 6), alongside STINGKO mice at young age (*n* = 12), adult age (*n* = 12), and old age (*n* = 6). Data are expressed as mean ± standard error of the mean (SEM). Statistical significance was assessed using an unpaired Student’s t-test, with ***p < 0.001

To assess potential locomotor impairments, mice were subjected to the Open Field Test (OFT). However, no statistically significant differences were found between the groups or across the different age groups (Fig. 3A-C). Therefore, our data suggest that STINGKO mice do not exhibit motor impairment, which would affect their ability to navigate the OFT arena during the observed period. To confirm this observation, we then performed the Rotarod test to assess locomotor performance and potential sedentary behavior. Our results showed no statistically significant differences in latency time between experimental groups across all ages evaluated (Fig. 3D-F). However, when analyzing running speed, a statistically significant difference was observed only at a young age between STINGKO and WT mice (Fig. 3G-I). STINGKO young mice exhibited a lower speed of 9 ± 0.55 rpm compared to 16 ± 0.87 rpm for WT young mice (Fig. 3G). When correlating this result with the findings from the hanging test, it can be inferred that STINGKO young mice likely experience greater difficulty supporting their body weight compared to WT young mice (Fig. 2D). These results suggest no underlying muscular or locomotor impairments that would indicate increased sedentarism or locomotor dysfunction in STING-deficient mice compared to WT mice.

### STING deficiency leads to the formation of lipid deposits in tissues

Subsequently, we assessed physical strength by the hanging test. The frequency of falls for each mouse was documented, and the mean values for each experimental group were illustrated (Fig. 2G-I). The incidence of falls escalated with age across all experimental cohorts. Throughout all evaluated ages, STINGKO mice demonstrated a greater incidence of falls in comparison to WT mice (Fig. 2G-J). The results indicate that STINGKO mice exhibit diminished capacity to maintain their grasp on the hanging test bars for prolonged periods. The diminished performance may be attributed to either a more significant locomotor deficit in STINGKO mice compared to WT mice or to the possible impact of body weight on their grip maintenance relative to WT controls.

Mice performed the Open Field Test (OFT) to evaluate potential locomotor deficits. No statistically significant differences were observed between the groups or among the various age categories (Fig. 3A-C). Consequently, our data indicate that STINGKO mice do not demonstrate motor deficits that would hinder their movement inside of the OFT arena within the studied timeframe. To validate this finding, we subsequently conducted the Rotarod test to evaluate locomotor ability and possible sedentary behavior. Our results indicated no statistically significant changes in latency time among experimental groups across all assessed age categories (Fig. 3D-F). A statistically significant difference in running speed was detected just at a young age between STINGKO and WT mice (Fig. 3G-I). STINGKO young mice demonstrated a reduced speed of 9 ± 0.55 rpm, in contrast to the 16 ± 0.87 rpm observed in WT young mice (Fig. 3G). Correlating this result with the findings from the hanging test suggests that STINGKO young mice likely encounter higher challenges in supporting their body weight compared to WT young mice (Fig. 2D). The results reveal the absence of muscle or locomotor deficits that could indicate enhanced sedentarism or locomotor dysfunction in STING-deficient mice relative to WT mice.

**Fig. 4:**
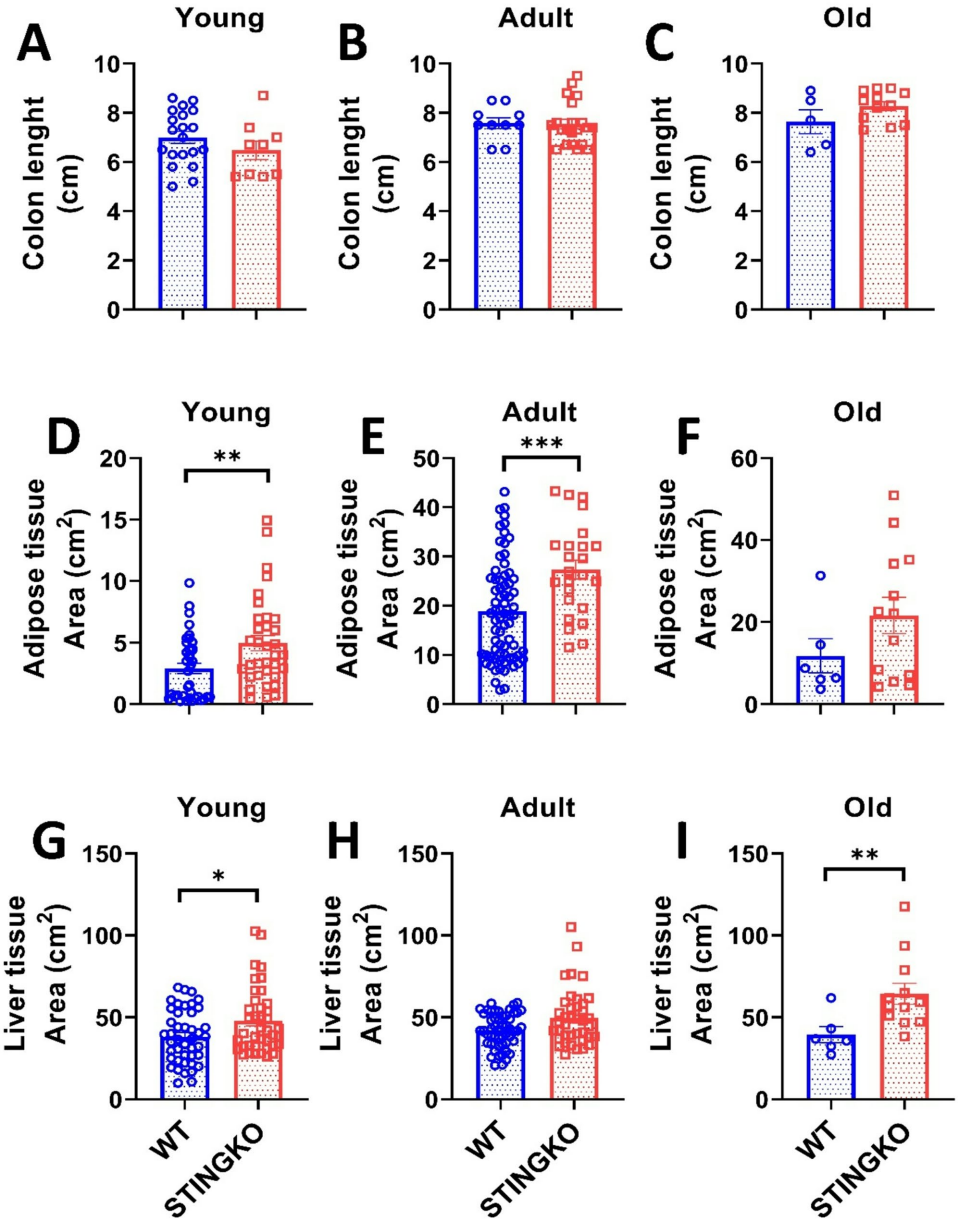
STING deficiency increases tissue area in metabolic tissues across different ages. Colon length in **A**) young (1–4 months), **B**) adult (10–15 months), and **C**) old (21–24 months) WT (blue) and STINGKO (red) mice. The experimental cohorts included WT mice at a young age (*n* = 20), adult age (*n* = 10), and old age (*n* = 5), with STINGKO mice at a young age (*n* = 9), adult age (*n* = 21), and old age (*n* = 12). Data are expressed as mean ± SEM. Statistical significance was assessed using an unpaired Student’s t-test, revealing no statistically significant differences. Adipose tissue area in WT and STINGKO mice at stages **D**) young, **E**) adult, and **F**) old. The experimental groups included WT mice at young age (*n* = 37), adult age (*n* = 72), and old age (*n* = 6), alongside STINGKO mice at young age (*n* = 34), adult age (*n* = 24), and old age (*n* = 13). Data are expressed as mean ± SEM. Statistical significance was assessed using an unpaired Student’s t-test, with *p ≤ 0.05 and ***p < 0.001. Liver regions in WT and STINGKO mice at **G**) young, **H**) adult, and I) old stages. The experimental cohorts comprised WT mice at young (*n* = 37), adult (*n* = 72), and old (*n* = 6) stages, alongside STINGKO animals at young (*n* = 34), adult (*n* = 24), and old (*n* = 13) stages. Data are expressed as mean ± SEM. Statistical significance was assessed using an unpaired Student’s t-test, with *p < 0.05

Animals subjected to a high-fat diet (HFD) demonstrate modifications in the digestive tract, characterized by an unchanged small intestine length (the location of fat absorption) and a reduction in colon length [31]. We assessed the colon length in STINGKO mice to ascertain any alterations. No notable variations were seen among the experimental groups (Fig. 4A-C), indicating that STING-deficient animals exhibit no diet-related, inflammatory, or gastrointestinal alterations [31].

Consequently, to determine the reason for the disparity in body weight between STINGKO and WT mice, we analyzed whether it resulted from increased fat storage. We quantified the gonadal adipose tissue of each experimental group and observed that STINGKO mice exhibited a substantial increase in adipose tissue area relative to WT mice at both young and adult stages (Fig. 4D-E). The same trend was noted in the old cohort; however, it lacked statistical significance (Fig. 4F). Additionally, we evaluated the liver area in both experimental groups to determine whether fat had accumulated in the hepatic tissue. In young STINGKO mice, an increase in liver area was seen as compared to WT mice (Fig. 4G). While a trend indicating enlarged liver size was noted in STINGKO animals during adulthood and old age relative to WT, this disparity lacked statistical significance (Fig. 4H-I). These findings indicate that the elevated body weight of STINGKO mice could be the consequence of increased lipid accumulation in both the liver and adipose tissue during the old and adult stages of life.

**Fig. 5:**
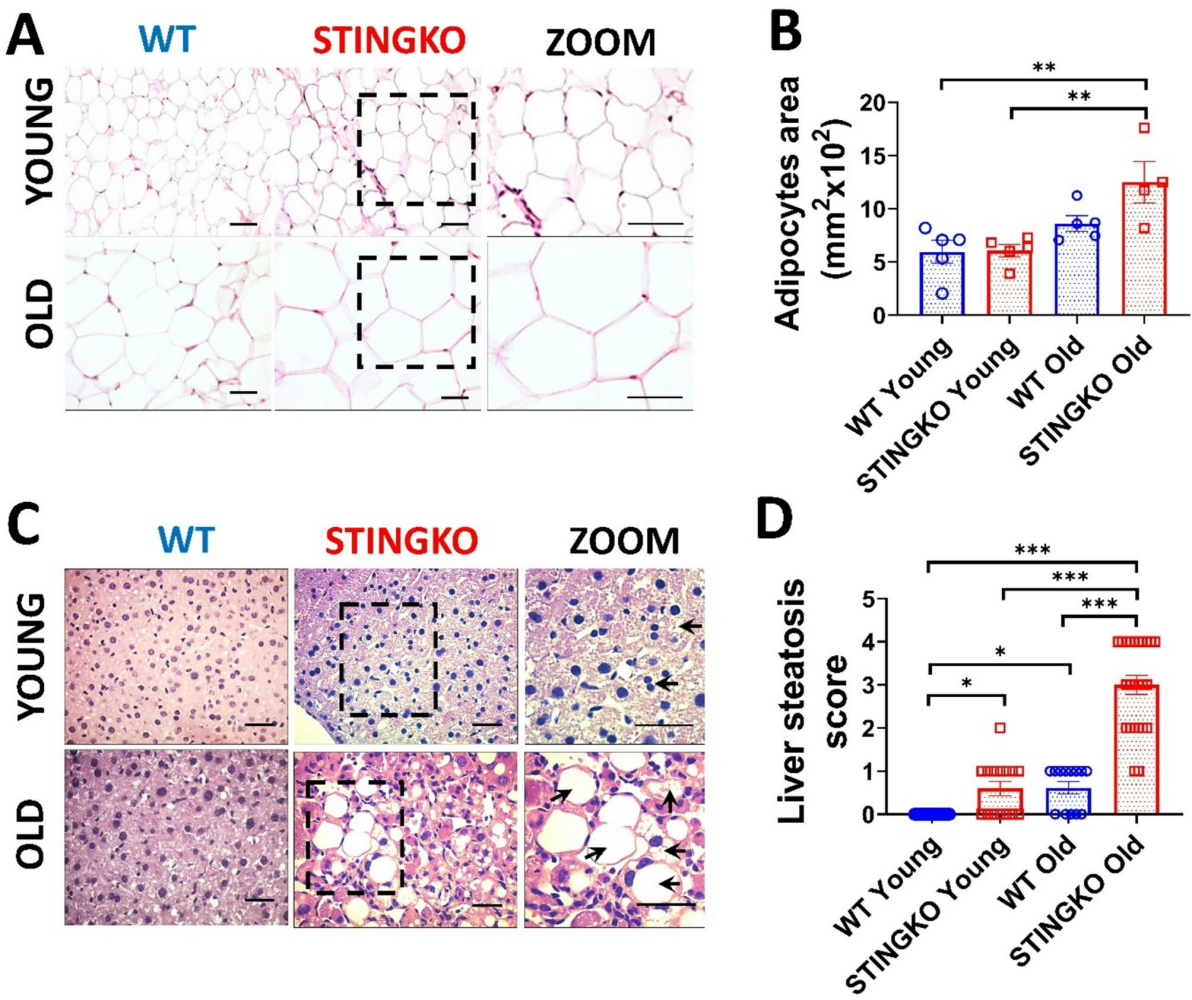
STING deficiency results in adipocyte hypertrophy and hepatic steatosis at multiple ages. Representative photographs from H&E staining of **A**) adipose tissue and **C**) liver sections from WT and STINGKO mice at young (1–3 months) and old (21–24 months) stages. The scale bar denotes 100 µm, but in the magnified images, it denotes 200 µm. The ‘zoom’ photos (in **A** and **C**) are enlarged representations of STINGKO animal photographs, with the expanded regions delineated by black boxes. WT (blue) and STINGKO (red) mice. In **B**) the measurement of adipocyte area. The experimental cohorts comprised WT mice at both young (*n* = 5) and old (*n* = 5) ages, in addition to STINGKO mice at young (*n* = 5) and old (*n* = 5) ages. Data are expressed as mean ± SEM and analyzed via the one-way ANOVA, with **p < 0.01. In **C**), back arrows signify fat accumulation within the hepatic tissue. In **D**) the steatosis index. The experimental groups comprised WT mice at a young age (*n* = 25) and an old age (*n* = 15), in addition to STINGKO mice at a young age (*n* = 15) and an old age (*n* = 24). Data are expressed as mean ± SEM and analyzed via one-way ANOVA, with *p ≤ 0.05 and ***p < 0.001

To ascertain that the increased adipose and hepatic tissue area in STINGKO mice results from heightened lipid accumulation, we examined the fat content in the parenchymal cells of each tissue. We utilized histological sections to assess the adipocyte area (Fig. 5A) and the degree of hepatic steatosis (Fig. 5C). In comparison to WT mice, STINGKO mice demonstrated a marked increase in adipocyte area or hypertrophy, especially in the older age cohort (Fig. 5A, B). Moreover, STINGKO mice exhibited hepatic steatosis at an earlier stage than WT mice (Fig. 5C, D). The results indicate greater deposits of lipids in the metabolic tissues of STINGKO mice relative to WT mice. The appearance of significant lipid droplets in the hepatocytes of juvenile STINGKO mice indicates an enhanced rate of lipid synthesis or storage in these subjects.

### STING deficiency results in elevated levels of triglycerides and total cholesterol

**Fig. 6:**
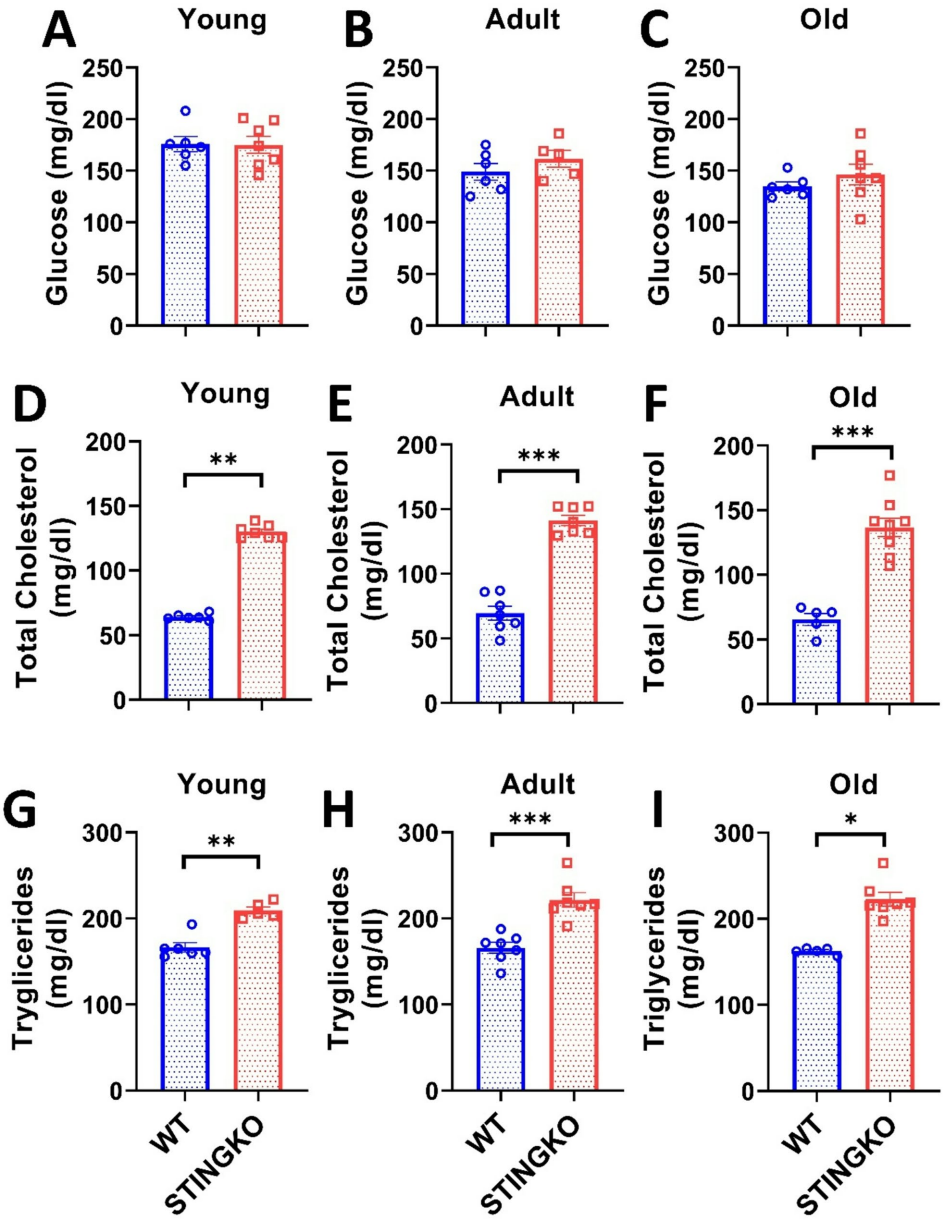
STING deficiency results in increased triglycerides and total cholesterol across different ages. Blood glucose levels were assessed in **A**) young (1–4 months), **B**) adult (10–15 months), and **C**) old (21–24 months) WT (blue) and STINGKO (red) mice. The experimental cohorts included WT mice at young (*n* = 6), adult (*n* = 6), and old (*n* = 6) stages, alongside STINGKO animals at young (*n* = 7), adult (*n* = 5), and old (*n* = 7) stages. Data are expressed as mean ± SEM. Statistical significance was assessed using an unpaired Student’s t-test, revealing no statistically significant differences. The total cholesterol levels in WT and STINGKO mice at developmental stages **D**) young, **E**) adult, and **F**) old. The experimental groups included WT mice at young age (*n* = 6), adult age (*n* = 7), and old age (*n* = 5), along with STINGKO mice at young age (*n* = 7), adult age (*n* = 7), and old age (*n* = 9). Data are expressed as mean ± SEM. Statistical significance was assessed using an unpaired Student’s t-test, with **p ≤ 0.01 and ***p < 0.001. The triglyceride levels at **G**) young, **H**) adult, and I) old stages. The experimental groups included WT mice at young age (*n* = 6), adult age (*n* = 7), and old age (*n* = 5), together with STINGKO mice at young age (*n* = 5), adult age (*n* = 7), and old age (*n* = 7). Data are expressed as mean ± SEM and evaluated via one-way ANOVA, with significance levels of *p ≤ 0.05, **p ≤ 0.01, and ***p < 0.001

To determine if the heightened fat accumulation in metabolic organs correlates with elevated circulating lipids in STINGKO mice, we assessed glucose, cholesterol, and triglycerides (Fig. 6). The measurements indicated no variations in circulating glucose levels between WT and STINGKO animals at any of the assessed ages (Fig. 6A-C). Notably, substantial variations were detected in cholesterol levels (Fig. 6D-F) and triglycerides (Fig. 6G-I). Total cholesterol levels in WT mice were around 64.02 ± 1.02, 69.37 ± 4.91, and 65 ± 4.74 mg/dl for the young, adult, and old age groups, respectively (Fig. 6D-F). Conversely, STINGKO mice demonstrated total cholesterol levels of around 130.07 ± 1.89; 141.4 ± 3.97, and 150.53 ± 6.87 mg/dl at the respective ages (Fig. 6D-F), suggesting that STING is involved in cholesterol metabolism. Upon evaluation of triglyceride levels (Fig. 6G-I), WT mice exhibited 166.40 ± 5.54; 165.97 ± 5.91; 162.46 ± 1.62 mg/dl at young, adult and old age categories, respectively, whereas STINGKO mice demonstrated average triglyceride levels of 209.36 ± 4.00; 221.40 ± 8.53; and 222.59 ± 8.53 mg/dl at young, adult, and old ages, respectively (Fig. 6G-I). The data indicate that in the absence of STING, the circulation levels of total cholesterol and triglycerides remain increased throughout life relative to wild-type mice.

## Discussion

Our findings indicate that STING deficiency leads to an increase in body weight in male STINGKO mice. The phenotype was assessed under standard dietary conditions and was not influenced by the amount of food consumed. The observed weight gain was not attributable to increases in muscle mass, strength, or locomotor changes across the age groups analyzed. It was found to correlate with the expansion of metabolic tissue, encompassing adipose tissue hypertrophy and liver steatosis. Despite stable glucose levels, STINGKO mice demonstrated increased circulating triglycerides and total cholesterol across all age groups. The findings indicate that lifelong STING deficiency facilitates lipid accumulation in tissues and circulation, independent of metabolic disorders. This contrasts with earlier research indicating that STING inhibition or deficiency in experimental obesity models decreases lipid levels and alleviates disease progression [5, 6, 8, 10, 13]. In contrast to studies that evaluate metabolic parameters at single time points after high-fat diet (HFD) exposure with total, incomplete, or transient STING activity inhibition, our research examines the lifelong effects of constitutive STING deficiency on lipid metabolism.

We examined metabolic parameters in mice deficient in STING expression across all tissues, including those with high metabolic activity. In contrast to our findings, STING^gt^ (Tmem173^gt^) mice, which have ER-localized STING that cannot translocate for signaling (https://www.jax.org/strain/017537), initially exhibited no differences in fat accumulation. After 12 weeks on a high-fat diet (HFD), STING^gt^ mice demonstrated lower body weight, decreased circulating fat, and reduced fat stores in comparison to wild-type mice [21]. In contrast, our study demonstrated that complete STING deficiency resulted in weight gain and increased triglyceride levels independent of HFD, indicating further metabolic functions of ER-localized STING. We propose that STING may modulate essential enzymes involved in triglyceride synthesis located in the ER membrane, specifically Glycerol Phosphate Acetyltransferase (GPAT) and Acylglycerophosphate Acyltransferase (AGPAT) [32, 33]. Additional research is required to validate STING’s interaction with these proteins or other ER components and to elucidate its specific role in triglyceride metabolism.

In addition to elevated triglycerides, STINGKO mice exhibited increased levels of total cholesterol alongside elevated triglycerides. STING contains cholesterol-binding sites, and its movement from the ER to the Golgi is contingent upon these sites [34]. STING translocation is inhibited in high cholesterol conditions, indicating a direct relationship between cholesterol levels and its translocation and signaling [34]. The absence of STING may disrupt this regulatory system, leading to cholesterol accumulation. STING is associated with cholesterol lysosome transport proteins, such as Niemann-Pick C1 protein (NPC1) [35]. This interaction indicates that STING deficiency may impair cholesterol homeostasis, leading to its accumulation and circulation in STINGKO mice. Our study did not investigate the potential influence of STING on lipogenesis; accordingly, future research should assess indicators of lipogenesis.

STING is significantly expressed in adipocytes, preadipocytes, and macrophages, with its activation in obesity models correlating with enhanced lipid accumulation [18, 21]. The results demonstrated that STING deletion promotes lipid accumulation, leading to hypertrophic adipocytes in STINGKO animals, regardless of obesity status and at an advanced age. This observation aligns with evidence from primary culture adipocytes subjected to STING inhibitors [36]. In vitro studies demonstrate that STING activation modulates autophagy in both mice and human adipocytes, while also promoting mitophagy through the detection of damaged mtDNA. Pharmacological suppression of STING influences autophagy, leading to a reduction in functional mitochondria, heightened inflammation, and accumulation of lipid droplets, independent of obesity [36]. This process may elucidate the late-onset lipid accumulation in adipocytes observed in our STINGKO animals. Age-related mitochondrial DNA damage, coupled with a lifetime deficiency in STING within adipocytes, is anticipated to impair STING-mediated regulation of lipid autophagy. This impairment may lead to lipid accumulation, hypertrophy, and potentially increased levels of pro-inflammatory cytokines. Future studies should examine mtDNA integrity and the levels of autophagy-related proteins in STINGKO mouse adipocytes to support this theory.

The expression of STING in the liver is still debated. While it is mostly found in Kupffer cells and liver macrophages [9]certain studies suggest that it also exists in hepatocytes, where it can cause damage and apoptosis [37]. In animal models, myeloid-specific STING and Yes-Associated Protein (YAP) knockouts enhanced HFD-induced liver steatosis, lipid accumulation, and triglyceride levels [38]. YAP, a protein induced by mitochondrial stress and liver injury [38, 39]has been shown to bind with STING in this tissue, with STING controlling YAP through autophagy under HFD or liver damage circumstances [38]. The HFD-induced lipid accumulation in these double-knockout mice’s hepatocytes was caused by defective autophagy and decreased macrophage-hepatocyte communication [38]. Similarly, the STINGKO mice utilized in our investigation exhibited liver steatosis, possibly due to age-related damage (e.g., damage to mtDNA) and possibly disrupting the STING-YAP axis, even in the absence of HFD, similar to the effects found in adipocytes.

STING activity and its involvement in adipose and liver tissues in obesity models have been extensively studied [5]. Nevertheless, the precise function of STING in the metabolism of human fat or metabolic human diseases is a relatively new field of research. For instance, human STING alleles, particularly HAQ and AQ, have distinct effects on fat metabolism and immunological tolerance due to distinctive amino acid changes (R71H-I229A-R292Q in HAQ and G230A-R293Q in AQ) [40]. The HAQ genotype, which is associated with increased fat storage and decreased cyclic dinucleotide detection, has a higher affinity for fatty acid desaturase 2 (FADS2). FADS2, an ER-resident enzyme, is required to desaturate dietary omega-3 and omega-6 polyunsaturated fatty acids (PUFAs). These FADS2-generated PUFAs have been demonstrated to inhibit STING activity, reducing IFN production [11, 40, 41]. While our current study did not examine IFN or pro-inflammatory molecules, future research should investigate these factors and fully describe the immune response in STINGKO mice to have a better understanding of their altered reactivity to injury or infection at different ages.

Recent studies indicate that STING is significantly expressed in immune cells and plays a critical role in their development and function [42]. Additional studies on STING deficiency across different tissues devoid of pathology have highlighted its essential role in cellular function over time [43]. Hopkins et al. documented tissue structural loss, impaired cell communication, and an aging-related gene expression profile in STINGKO mice aged 3 to 24 months, aligning with the long-term alterations we observed [44]. In contrast, their histological analysis of the liver indicated structural changes without the presence of hepatic steatosis at the advanced age [44]which differs from our findings. The observed difference may result from unspecified dietary variations and a smaller sample size (*n* = 4–5 compared to our *n* = 15–25) [44]. The role of tissue-resident macrophages [44] in structural disruption is emphasized, particularly in relation to Kupffer cells in the liver and adipose-resident macrophages, which display elevated STING levels [9, 19]. We propose that STING in these cells plays a crucial role in metabolism, facilitating lipid accumulation over time. The findings indicate that the function of STING varies with age, potentially affecting macrophages [44]. This underscores the necessity of incorporating age as a factor in studies and therapeutic approaches related to STING and metabolic diseases. The long-term role of STING in metabolic tissues is not well understood [45]in contrast to its established age-related increase in nervous tissue associated with neurodegeneration [45–48]. Gulen et al. reported no changes in body weight in aged WT mice (19–20 months) following short-term STING pharmacological inhibition (13 days with H-151). This finding contrasts with our results [43]. The brief inhibition in aged mice may differ significantly from our study’s lifelong STING deficiency, potentially accounting for the observed discrepancies.

Obesity accelerates the aging process by inducing macromolecular damage and disrupting functional proteins, which results in cellular and tissue dysfunction [49]. The STINGKO mice demonstrated increased blood lipid levels and mild steatosis as early as 1–3 months, consistent with prior reports on STINGKO mice of the same age, but solely under HFD conditions [17]. Consequently, the potential exists that early lipid elevation may expedite aging and play a role in metabolic dysfunction in STINGKO mice. This may establish a detrimental cycle in which STING deficiency leads to lipid accumulation, subsequently accelerating premature aging and exacerbating tissue damage. Future research should evaluate aging markers in these mice to elucidate this relationship.

The increase in fat accumulation in STINGKO mice is notably exacerbated with age, indicating a potential interaction between STING deficiency and age-related hormonal changes. Aging correlates with reductions in anabolic hormones, including sexual hormones, growth hormone, and IGF-1 ^50^, alongside elevated levels of catabolic and stress-related hormones like glucocorticoids [50, 51]. These hormonal changes contribute to fat redistribution and ectopic lipid deposition, especially in the liver and visceral areas [50, 51]. The lack of STING may exacerbate or inadequately counteract these endocrine changes, thus increasing vulnerability to metabolic dysregulation in older animals. These findings collectively indicate a significant role for STING in the preservation of endocrine-metabolic homeostasis throughout the lifespan [17]. Additional research on hormone levels, receptor expression, and downstream signaling in STINGKO male and female mice across various ages is crucial for clarifying the mechanisms that connect STING, aging, and lipid metabolism.

Changes in hormonal regulation outside of traditional inflammatory pathways may partially account for the heightened adiposity seen in STINGKO mice. The STING pathway is associated with metabolic inflammation and insulin signaling [21]; its deficiency may interfere with the inflammatory balance necessary for adequate insulin sensitivity, potentially leading to lipid accumulation in peripheral tissues [52]. Additionally, STING may have an indirect effect on the hypothalamic–pituitary–adrenal (HPA) axis, leading to changes in corticosterone levels [53]which are associated with promoting lipogenesis and fat deposition [54, 55]. Dysregulation of adipokines, including leptin and adiponectin, which are essential for insulin sensitivity, may contribute to the observed phenotype [4, 56]. Despite stable glucose levels in STINGKO mice, hormonal imbalances may contribute to the elevated triglyceride levels and hepatic steatosis observed [57, 58]suggesting that STING may have a more extensive role in endocrine-metabolic homeostasis.

On the other hand, a study investigated the impact of HFD duration on metabolic dysfunction-associated steatosis liver disease (MASLD) utilizing STING^gt^ mice [59]. STING^gt^ mice demonstrated protection following 3 months of HFD; however, this advantage diminished after 7 months, resulting in fat accumulation similar to that observed in WT mice [59]. This indicates that prolonged HFD-induced obesity modifies the function of STING, even in its inactive state, thereby reversing previous protective effects. These changes may arise from damage induced by lipid accumulation or the advanced age of the mice at the conclusion of the study. Future research should evaluate the regulation of STING in fat metabolism across various ages in both obese and non-obese individuals to elucidate the effects of aging.

Finally, more research is needed to identify whether STING deficiency during early development causes structural abnormalities and premature aging in metabolic tissues, resulting in lipid buildup. Our findings indicate that STING plays a complicated role in metabolic control across the lifespan, regulating lipid homeostasis beyond previously identified processes. We believe that age, pathological length (chronic vs. acute), and treatment timing all play important roles in evaluating STING as a therapeutic target for obesity and metabolic disorders. This emphasizes the need to study the levels, activity, and localization of the STING pathway in various cell types across the lifespan in metabolic tissues. Given the serious consequences of constitutive STING deficiency or long-term suppression, caution should be exercised when contemplating STING modification as a therapeutic method in metabolic illnesses, including obesity.

## Conclusions

The findings suggest that STING is essential for the regulation of fat storage across the lifespan in mice, especially within metabolic tissues. Results present new data indicating that chronic STING deficiency results in elevated lipid accumulation independent of a high-fat diet or metabolic disorders, implying previously unrecognized regulatory functions of STING in lipid metabolism. Future research should examine the differential expression of STING across specific cell types within these tissues to enhance our comprehension of its function in a cell-type-specific context.

## Data Availability

The datasets used and/or analyzed during the current study are available from the corresponding author upon reasonable request.
